# Editorial: Advances in generative artificial intelligence for mental health

**DOI:** 10.3389/fdgth.2026.1868217

**Published:** 2026-06-04

**Authors:** Nuo Han, Zengda Guan, Ang Li, Xiaoqian Liu, Xingyun Liu, Jia Xue

**Affiliations:** 1 Bay Area School of Applied Psychological Sciences, Beijing Normal University, Zhuhai, China; 2 Beijing Key Laboratory of Applied Experimental Psychology, National Demonstration Center for Experimental Psychology Education (Beijing Normal University), Faculty of Psychology, Beijing Normal University, Beijing, China; 3 Department of E-commerce, School of Business, Shandong Jianzhu University, Jinan, China; 4 Department of Psychology, Beijing Forestry University, Beijing, China; 5 State Key Laboratory of Cognitive Science and Mental Health, Institute of Psychology, Chinese Academy of Sciences, Beijing, China; 6 Department of Psychology, University of Chinese Academy of Sciences, Beijing, China; 7 Key Laboratory of Adolescent Cyberpsychology and Behavior, Ministry of Education, Wuhan, China; 8 Key Laboratory of Human Development and Mental Health of Hubei Province, School of Psychology, Central China Normal University, Wuhan, China; 9 Factor Inwentash Faculty of Social Work, University of Toronto, Toronto, ON, Canada; 10 Faculty of Information, University of Toronto, Toronto, ON, Canada

**Keywords:** challenges, digital mental health, generative artificial intelligence, implementation, opportunities

## Introduction

Generative artificial intelligence (GAI) is rapidly reshaping the landscape of digital mental health. Unlike earlier forms of artificial intelligence (AI) that were designed primarily for classification, prediction, or decision support, GAI systems can generate language, images, simulated cases, therapeutic dialogues, and context-sensitive recommendations (1). This generative capacity creates new possibilities for mental health assessment, intervention, training, supervision, and service delivery (2). At the same time, it raises urgent questions about safety, reliability, clinical accountability, regulation, and the appropriate boundaries between human and machine roles in mental healthcare (3, 4).

This Research Topic, *Advances in Generative Artificial Intelligence for Mental Health*, brings together new work on the opportunities and challenges of applying GAI in mental health. The contributions collectively reflect a field moving beyond general enthusiasm toward more concrete questions: What can GAI systems actually do in mental health contexts? Under what conditions are they useful? Where do they fail? How should clinicians, developers, policymakers, and service users govern their deployment?

Several contributions address the potential of GAI and related AI systems to support clinical work. Mircea et al. examined AI-driven decision support in mental health assessments and its association with clinician resilience and preparedness, highlighting the potential role of AI tools in reducing informational burden and supporting service delivery. Ruan et al. proposed the “augmented clinician” as a framework for human–AI collaboration, emphasizing that AI should not replace clinical judgment but may extend clinicians' capacity when embedded within appropriate professional, ethical, and organizational safeguards. Together, these studies suggest that the future of GAI in mental healthcare should be understood less as the automation of care and more as the careful redesign of human–AI systems.

Other articles focus on intervention and user-facing applications. Mo et al. developed a GPT-4-based problem-solving therapy chatbot for young individuals in the post-COVID-19 era, illustrating how large language models (LLMs) can be adapted for structured self-help interventions. Cui et al. developed and evaluated an LLM-based suicide intervention chatbot, addressing one of the most clinically sensitive areas for digital mental health tools. These studies point to the promise of scalable, accessible, and personalized support, particularly for populations facing barriers to traditional services. However, they also emphasize that mental health chatbots require rigorous evaluation, careful risk management, and explicit escalation pathways when users present with severe distress or crisis-related issues.

This Research Topic also includes work on the technical infrastructure needed to advance responsible research. Warner et al. introduced a synthetic patient and interview transcript creator as a tool for developing and evaluating LLMs in mental health. Such an approach may help address persistent barriers involving privacy, data scarcity, and the difficulty of obtaining high-quality clinical interaction data. Nevertheless, synthetic data should be treated as a complement rather than a substitute for real-world validation. Its usefulness depends on the fidelity, representativeness, and clinical plausibility of the generated cases.

The social, ethical, and policy dimensions of GAI are also a prominent theme in this collection. Hipgrave et al. examined clinicians' perspectives on the use of generative AI chatbots in mental healthcare, identifying both perceived benefits and concerns. Refoua et al. argued that general-purpose generative AI is already functioning as a de facto mental health provider for some users, creating an urgent need for clearer regulation. Bélisle-Pipon further warned that generative AI may foster therapeutic misconception among vulnerable users, especially when systems simulate empathy, authority, or therapeutic competence without the accountability and contextual understanding of trained professionals. These contributions underscore a central tension: GAI systems may expand access to support, but they may also blur the boundaries between information, companionship, coaching, and clinical care.

The collection also broadens the discussion beyond clinical intervention. Xu et al. investigated how topic content shapes personality-tailored persuasion by LLMs, raising important implications for personalized mental health communication. Persuasive capacity can be beneficial when used to promote help-seeking, adherence, or psychoeducation, but it also demands careful governance to prevent manipulation, over-dependence, or inappropriate influence.

Taken together, the articles in this Research Topic suggest several priorities for the next stage of research. First, empirical evaluations must keep pace with technological innovations. Studies should assess not only usability and acceptability but also clinical effectiveness, adverse effects, equity, and long-term outcomes. Second, GAI tools should be designed around clear use cases and risk levels. Low-risk psychoeducation, clinician documentation support, crisis triage, and therapeutic dialogue require different standards of evidence and oversight. Third, human oversight must be specified rather than assumed. The field needs more precise models indicating when clinicians, caregivers, peers, or emergency services should enter the loop. Fourth, governance frameworks should address transparency, privacy, bias, liability, and user understanding of system limitations.

GAI is unlikely to be a simple solution to the global mental health burden. Its value will depend on whether it can be integrated into mental health systems in ways that are clinically meaningful, ethically defensible, and socially equitable. This Research Topic contributes to this agenda by documenting both the promise and the risks of GAI in mental health. The central task now is not only to build more capable systems but also to ensure that these systems are evaluated, regulated, and implemented in ways that protect users while strengthening human-centered mental healthcare.

The author(s) declared that they were an editorial board member of Frontiers at the time of submission. This had no impact on the peer review process and the final decision.
